# Antibiotic carry over is a confounding factor for cell-based antimicrobial research applications

**DOI:** 10.1038/s41598-025-14186-7

**Published:** 2025-08-03

**Authors:** Miran Yousri Elfar, Helen Louise Brown, Aled Clayton, Phil Stephens

**Affiliations:** 1 School of Dentistry, Cardiff University, Health Park Campus, Cardiff, CF14 4XY UK; 2 School of Biosciences, Cardiff University, Sir Martin Evans Building, Cardiff, CF10 3AX UK; 3 Division of Cancer & Genetics, School of Medicine, Tenovus Building, Heath Park, Cardiff, CF14 4XN UK

**Keywords:** Chronic wound, Chronic wound infection, Fibroblast, *Staphylococcus aureus*, Biofilm, Antimicrobials, Bacteriology

## Abstract

Chronic wounds often host pathogens like *Staphylococcus aureus*, prompting interest in developing new antimicrobial and wound healing strategies, including the utilisation of extracellular vesicles (EVs). Whilst there has been a recent emphasis within the EV community to ensure standardization of characterization and isolation techniques, there has been less focus placed on the upstream tissue culture methodologies used for collection of vesicle-containing conditioned medium (CM). Hence, this study investigated the antimicrobial properties of the CM used for EV enrichment. CM exhibited bacteriostatic effects against penicillin-sensitive *S. aureus* NCTC 6571, but not penicillin-resistant *S. aureus* 1061 A. Further analysis revealed that the antimicrobial activity was due to residual antibiotics rather than cell-secreted factors, specifically the retention and release of penicillin to tissue culture plastic surfaces. Pre-washing cells and minimizing antibiotic concentrations in basal medium reduced this carry-over effect. These findings emphasize the importance of controlling antibiotic use in tissue culture to avoid misleading conclusions about the antimicrobial potential of CM or EVs. Researchers should carefully consider medium selection and supplementation during method development as accurately determining the antimicrobial mechanisms of any CM is essential for validating future cell-based therapeutic applications.

**Supplementary Information:**

The online version contains supplementary material available at 10.1038/s41598-025-14186-7.

## Introduction

Antimicrobial resistance (AMR) is a global threat and causes significant morbidity and mortality. Recent estimates predict that AMR will continue to rise, with those over 70 most at risk and the largest increase in AMR-associated mortality expected within South East Asia, Latin America and the Caribbean^1^. Nosocomial opportunistic infections are often caused by members of the normal microbiome, including *Staphylococcus aureus*, with wound infection comprising a third of all nosocomial infections^2^. Within chronic wounds, *S. aureus* and coagulase-negative *Staphylococcus* species, such as *Staphylococcus epidermidis*, are commonly isolated from wounds, identified both within wounds clinically diagnosed as infected (symptomatic infection) and those with no clinical symptoms of infection (asymptomatic)^3,4^. Studies suggest that AMR genes are common within *S. aureus* isolates originating from wounds^5–7^, with significant effort now being focused on developing novel antimicrobial strategies to combat these infections.

Against this background, the therapeutic potential of extracellular vesicles (EVs) and cell secreted products is increasingly being recognized, in varied contexts. Host-derived EVs are able to stimulate reparative processes and have also been shown to have antimicrobial properties^8^. Indeed, much has previously been reported on the antimicrobial and wound healing properties of stem cell EVs^9–11^. The field of EV research has exploded in popularity over the last decade^12^, with many research groups realizing the potential of EVs, originating from both the host and infectious agents, as biomarkers, therapeutics or biological drivers of pathology. Whilst there has been a recent emphasis within the EV community to ensure standardization of characterization and isolation techniques (summarized recently in^13,14)^:, there has been less focus placed on the upstream tissue culture methodologies used for collection of vesicle-containing conditioned medium (CM). Antibiotic supplements are known to be included in many studies in which tissue culture is required as an initial step prior to collecting materials for downstream processing, such as when isolating EVs.

Although it is widely accepted that the inclusion of antimicrobials within tissue culture system can sometimes lead to off-target effects, often other considerations drive researchers to include them within their experimental models. Tissue culture media are a rich and complex source of nutrients which can support the growth of both the cells they are designed to support, as well as a wide range of microbial species^15^. Whilst good aseptic technique and the use of biological safety cabinets can minimize the chances of any microbes being introduced into the tissue culture by the operator, the methods are nonetheless not foolproof. For example, in primary cell culture, or when large volumes are required, antibiotic/antimycotic inclusion within the medium can be particularly useful to suppress the growth of any microbial contaminants.

Penicillin and streptomycin, in a dual solution (referred to hereafter as PenStrep) or further combined with the antimycotic reagent amphotericin B (combined solutions of penicillin, streptomycin and amphotericin B are referred to hereafter as AA), are a common choice of antibiotics to include in routine tissue culture^16,17^. Whilst antibiotics and antimycotics reduce the possibility of microbial contamination, it has long been known that they can alter the phenotypic characteristics of cells. Addition of gentamicin to several breast cancer cell lines increased the production of reactive oxygen species and subsequent DNA damage^16^. Transcriptomic analysis of the liver cell line HepG2 reported that 209 genes were differentially expressed in the presence of PenStrep^18^. Genes with altered expression included several transcription factors, suggesting that gene transcription alterations in the presence of PenStrep is likely to be widespread and influence multiple pathways. The inclusion of PenStrep in tissue culture medium altered the action and field potential of cardiomyocytes^19^ as well as the electrophysiological properties of hippocampal pyramidal neurones^20^, highlighting its potential to affect experimental outcomes. It should be noted that information on the impact of antimicrobials on tissue culture systems is by no means a new finding, with the work of Metzger et al.^21^ in 1954 demonstrating that fibroblast growth was reduced in the presence of the tetracycline antibiotic, Terramycin, and complete inhibition of growth was observed at concentrations of over 3000 µg/ml. Furthermore, when preparing EV stocks, many researchers use PenStrep as part of their routine cell maintenance protocols even if the PenStrep is absent during the medium EV conditioning step^22–26^.

The basis of this manuscript originated from unanticipated findings, when it was noted that CM collected for downstream EV enrichment, demonstrated antimicrobial activity against *Staphylococcus aureus* isolate NCTC 6571 but not to isolates found to have penicillin resistance. The activity was widespread across multiple EV-donor cells lines, and as such it was investigated if this effect was mediated by EVs or potentially due to the inclusion of antimicrobials within the routine tissue culture regimen.

## Results

### CM produced from different cell lines show antimicrobial activity

**Fig. 1:**
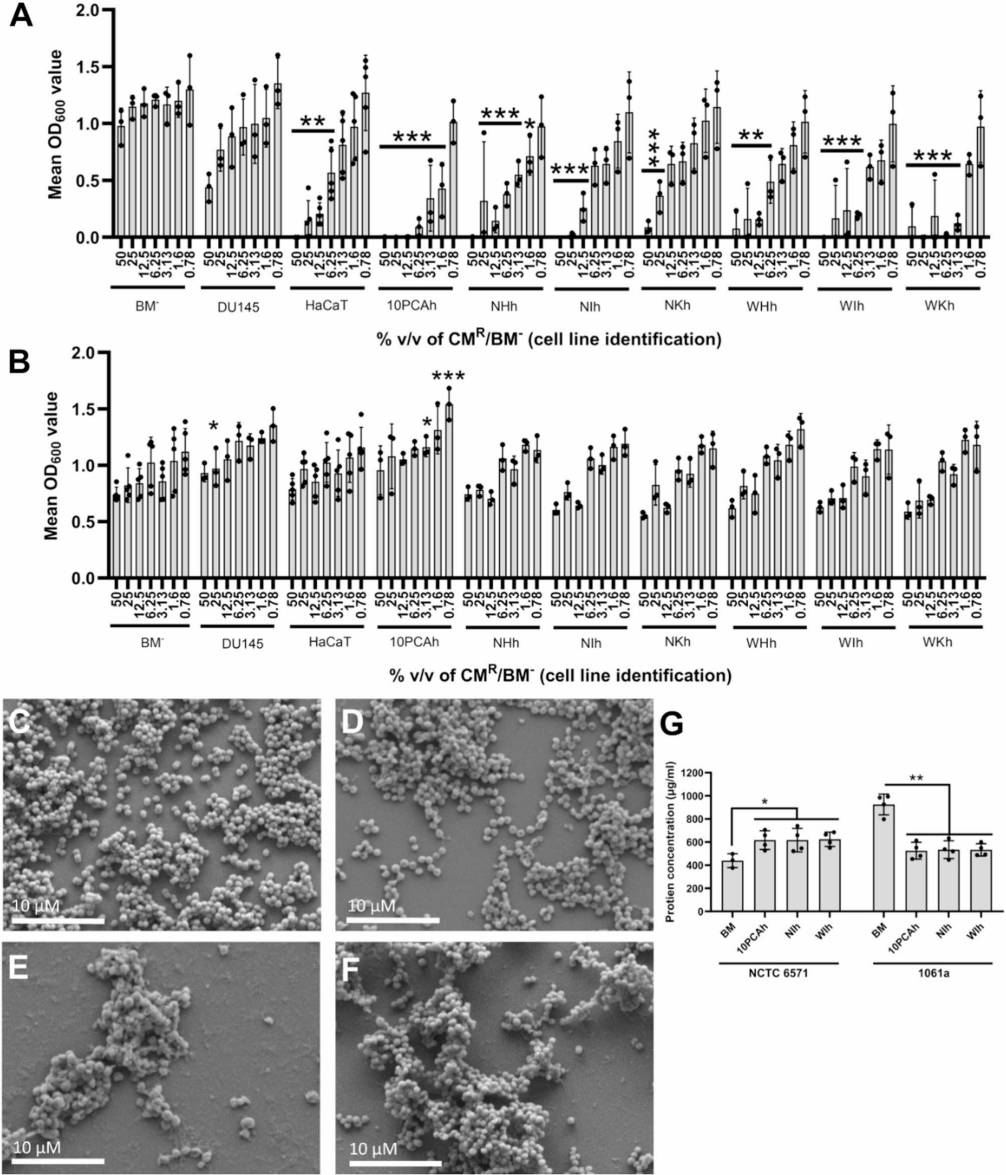
CM^R^ collected from a wide range of cell types showed antimicrobial activity against *S. aureus* NCTC 6571, but not the penicillin-resistant clinical isolate *S. aureus* 1061 A. **(A)** Growth of *S. aureus* NCTC 6571, **(B)** and *S. aureus* 1061 A was determined in the presence of the CM^R^ of nine cell lines and the basal (unconditioned) serum and antibiotic-free medium (BM^−^). **(C-F)** SEM images of 24 h biomass of *S. aureus* NCTC 6571 **(C)** and *S. aureus* 1061 A **(D)** in the presence of unconditioned BM^−^, or *S. aureus* NCTC 6571 **(E)** or *S. aureus* 1061 A **(F)** in 50% v/v WIh CM^R^. **(G)** Protein secretion into the bacterial supernatant was measured to determine potential loss of bacterial cell wall integrity and leakage of protein from the bacterial cells into the surrounding supernatant. All bars show mean values and error bars represent standard deviation, *N* = 3. Statistical significance was measured by Tukey test following one-way ANOVA (*, *P* < 0.05; **, *P* < 0.01; ***, *P* < 0.001). SEM images are representative, scale bar = 10 µM.

Initially CM was collected from a number of cell lines to assess potential antibacterial activity. The cell lines included within this study are listed in Table 1 and included dermal fibroblast cell lines derived from the healthy skin of three patients (named NHh, NIh and NKh), three patient-matched dermal fibroblast cell lines derived from venous leg ulcers from the same patients (named WHh, WIh and WKh), and a spontaneously immortalized human keratinocyte cell line (HaCaT). These seven cell lines were included as they are relevant to chronic wound research and both keratinocytes and fibroblasts are reported to display antimicrobial activity. An immortalized human oral mucosal lamina propria-progenitor cell (named 10PCAh) was included as previous data from the group also showed evidence that the CM from these cells was antimicrobial^27^. Finally, an epithelial cell line derived from a brain metastasis of a patient with advanced prostate cancer (DU145) was included as a negative control with no previously reported antimicrobial activity. Preparation of this CM included an initial (48 h) incubation of the cell monolayer in 1% v/v AA containing basal medium (BM^+^), before switching to AA and FBS free basal medium for the second (72 h) conditioning step (hereafter this CM is referred to as Routine CM or CM^R^). At concentrations of 50% down to 6.25% v/v, CM^R^ from all nine different cell lines demonstrated significant bacteriostatic activity against penicillin sensitive *S. aureus* NCTC 6571 relative to the appropriately diluted AA free basal medium (BM^−^) control (Fig. 1A; *P* ≤ 0.05, *N* = 3). However, the nine CM^R^ did not show the same growth inhibition effects against the penicillin resistant clinical isolate *S. aureus* 1061 A (Fig. 1B; *P* ≥ 0.05, *N* = 3). A wider screen of staphylococcal isolates also showed the same trend, with the growth of penicillin-susceptible isolates statistically significantly reduced at higher CM^R^ concentrations (Supp. Figure 1A to H; *P* ≤ 0.05, *N* = 3).

**Table 1:** A list of cell lines/bacterial strains, their origin and maintenance/growth conditions used within this study.

Human immortalised cell lines	Origin	Routine growth medium/conditions	Reference
10PCAh	Immortalised human oral mucosal lamina propria-progenitor cells	DMEM, 10% FBS, 1% L glutamine, 1% Antibiotic/Antimycotic 37 °C + 5% CO_2_	^29^
NHh	Immortalised human fibroblasts from healthy leg tissue (patient H)		^53^
NIh	Immortalised human fibroblasts from healthy leg tissue (patient I)		^53^
NKh	Immortalised human fibroblasts from healthy leg tissue (patient K)		^53^
WHh	Immortalised human fibroblasts from chronic venous wound tissue (patient H)		^53^
WIh	Immortalised human fibroblasts from chronic venous wound tissue (patient I)		^53^
WKh	Immortalised human fibroblasts from chronic venous wound tissue (patient K)		^53^
HaCaT	Spontaneously transformed human keratinocyte cell line		^54^
DU145	Prostate carcinoma cell line		^55^
**Bacterial isolates/strains**	**Origin (penicillin resistance status)**	**Bacterial growth medium/conditions**	**Reference**
*S. aureus* NCTC 6571	Lab strain susceptible to penicillin	TSA, TSB, MHB, BHI 35 °C/37°C ± 5% CO_2_	~In house
*S. aureus* 1061 A	Clinical strain resistant to penicillin		^56^
*S. aureus* 1004 A	Clinical strain resistant to penicillin		^56^
*S. aureus* 1038B	Clinical strain susceptible to penicillin		^56^
*S. aureus* NCTC 4137	Lab strain susceptible to penicillin		~In house
*S. aureus* NCTC 7791	Lab strain susceptible to penicillin		~In house
*S. aureus* EMRSA-15	Clinical strain resistant to penicillin		~In house
*S. epidermidis* 1064 A	Clinical strain resistant to penicillin		^56^
*S. epidermidis* ATCC 12,228	Lab strain susceptible to penicillin		~In house
*S. warneri* NCTC 5955	Lab strain susceptible to penicillin		~In house

To investigate the effects of CM^R^ on bacterial attachment, scanning electron microscopy was undertaken. Compared to BM^−^ (Fig. 1C) *S. aureus* NCTC 6571 biomass was notably reduced on exposure to CM^R^ at 50% v/v (Fig. 1E) whilst for *S. aureus* 1061 A attachment was still evident (Fig. 1D) compared to control (Fig. 1F). Total protein secreted by the two bacteria after being treated with different CM^R^ (10PCAh, NIh and WIh; Fig. 1G, *N* = 3) was also assessed. It was demonstrated that the total protein content of the bacterial supernatants of *S. aureus* NCTC 6571 treated with CM^R^ significantly increased relative to the control protein content (*P* < 0.05), whilst the bacterial supernatants of *S. aureus* 1061 A showed a significant lowering of protein content relative to the control (*P* < 0.01). Taken together these data indicate that *S. aureus* NCTC 6571, but not *S. aureus* 1061 A were susceptible to killing by cell produced CM^R^ and the antimicrobial activity observed in *S. aureus* NCTC 6571 appears to perturb cell integrity, causing leakage of cellular materials into the extracellular milieu. While the CM from all the cell lines (with the exception of DU145) showed statistically significantly reduced the growth of *S. aureus* NCTC 6571 at CM^R^ concentrations of 12.5% v/v and above, the 10PCAh cell line CM showed the highest inhibition and was so used in all subsequent experiments.

### Cellular confluency, pre-washing and sampling time point affect the antimicrobial activity of the CM^R^ against *S. aureus* NCTC 6571

**Fig. 2:**
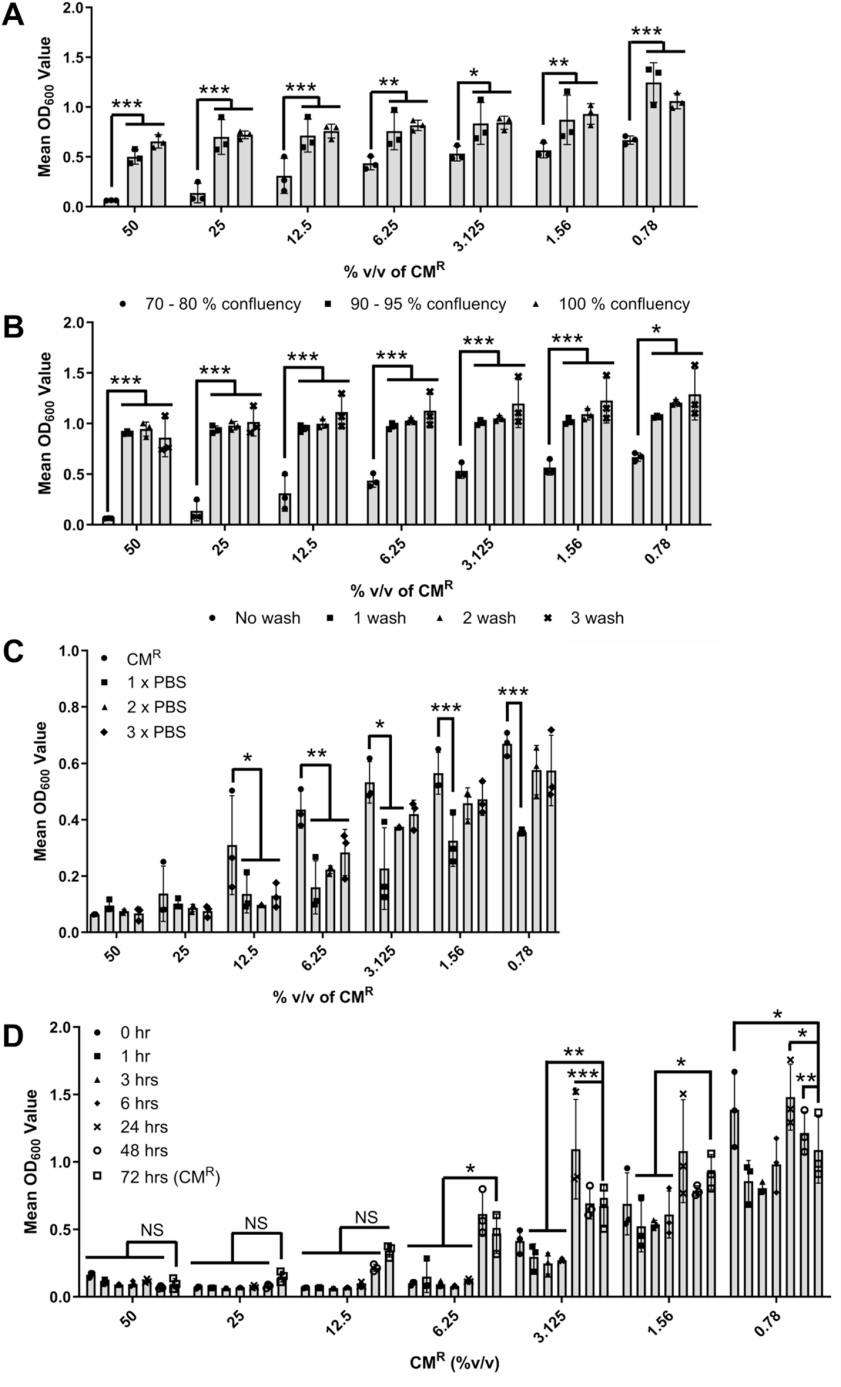
10PCAh CM^R^ demonstrated antimicrobial activity against *S. aureus* NCTC 6571 which could be ameliorated and/or reduced under certain conditions. **(A)** Increased the confluency of 10PCAh cells from 70–80% (black circles), 90–95% (black squares) and finally 100% confluency (black traingles) led to a reduction in the antimicrobial potency of the collected CM^R^. **(B)** Washing of cell monolayers once (black squares), twice (black triangle) or three times (black cross) after seeding, but prior to the commencement of the medium conditioning step (antibiotic-free medium), reduced antimicrobial potency of CM^R^ compared to the unwashed monolayer (black circle) control. **(C)** The collected PBS washings from **(B)** demonstrated antimicrobial activity similar to CM^R^. **(D)** CM^R^ samples collected at time intervals of 0 h., 3 h., 6 h., 24 h. and 48 h. all show statistically same antimicrobial activity to CM^R^ (72 h.) at concentrations of 12.5% v/v or above. All bars show mean values and error bars represent standard deviation, *N* = 3. Statistical significance was measured by Dunnett’s test following two-way ANOVA (*N* = 3) *, *P* < 0.05; **, *P* < 0.01; ***, *P* < 0.001).

The effect of cellular confluency at point of CM^R^ collection, and hence the amount of ‘uncovered’ tissue culture plastic, was investigated in relation to the antimicrobial activity of CM^R^. As demonstrated in Fig. 2A, as the cellular confluency increased (70–80% to 90–95% to > 100%), the antimicrobial activity of the collected CM^R^ decreased significantly regardless of the dilution of the CM^R^ utilized (*P* < 0.001, *N* = 3). This suggested that the antimicrobial factor could be something that was retained on the plastic rather than something actually secreted by the cells themselves. Next, pre-washing of the cell cultures, completed prior to the addition of the BM^−^ to the 70–80% confluency cells and subsequent 72-hour conditioning incubation was investigated (Table 2). Even after only one pre-wash of the cells, the antimicrobial activity of the subsequently collected CM^R^ was effectively removed (Fig. 2B; *P* < 0.001, *N* = 3), with this antimicrobial activity then presented in the sterile PBS wash solutions collected following each washing of the monolayer (Fig. 2C; *P* < 0.001, *N* = 3). Finally, the influence of the CM^R^ collection time point was investigated (0, 1, 3, 6, 24, 48 and 72 h; Fig. 2D). At CM^R^ concentrations of 12.5% v/v and higher no significant difference was observed between the CM^R^ collected, as was routine, at 72 h and those CM^R^ collected at earlier time points, even at zero hr. (medium added to the cells and then removed immediately). At all % v/v concentrations, the CM^R^ samples collected at timepoints of 0 to 6 h. showed equivalent or statistically improved biomass reduction (*P* ≤ 0.05, *N* = 3) than the CM^R^ collected at 72 h. This demonstrates that antimicrobial activity was not due to the secretion of factors from the CM^R^-conditioning cells.

**Table 2:** Different conditioning processes used to optimize the bacteriostatic activity of the collected condition medium (CM) and detect antibiotic carryover if any. Unless otherwise stated, advance binding tissue culture flasks (plastic 1) were used for the generation of all CM types (CM^R/P/T^).

Experimental procedure name	Cell seeding density (cell/ml)	Incubation 1		Interim processing	Incubation 2		Post collection processing
		Time (hr)	Medium		Time (hr)	Medium	
Routine CM (CM^R^)	4000	48	BM^+^	None	72	BM^−^	0.22 µM PES filtration Long-term storage at −80 °C
Placebo CM (CM^P^)	0	48	BM^+^	None	72	BM^−^	
Variable confluency	4000 (70–80%)	48	BM^+^	None	72	BM^−^	
	8000 (90–95%)						
	10,000 (100%)						
Monolayer wash	4000	48	BM^+^	1 x PBS wash	72	BM^−^	
				2 x PBS wash			
				3 x PBS wash			
AA concentration variation	4000	48	BM^+^ (0% AA)	None	72	BM^−^	
			BM^+^ (0.01% AA)				
			BM^+^ (0.1% AA)				
			BM^+^				
Collection time	4000	48	BM^+^	None	0	BM^−^	
					1		
					3		
					6		
					24		
					48		
					72		
Vessel type	4000 (Standard Treated vessel)	48	BM^+^	None	72	BM^−^	
	4000 (non-treated Polystyrene)						
	4000 (polypropylene)						

### Antibiotic concentration affects the antibacterial nature of cellular CM

**Fig. 3:**
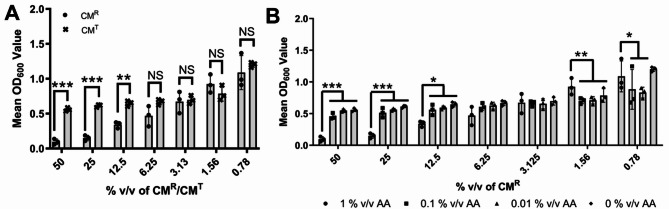
The antimicrobial activity of 10PCAh CM^R^ was due to AA carry over, not secretion of an antimicrobial molecule by the mammalian cells. **(A)** Antimicrobial activity against *S. aureus* NCTC 6571 is statistically significantly reduced (*P* ≤ 0.01) in CM^T^ (black cross) compared to CM^R^ (black circle). CM^T^ was generated without using AA at any stage of the CM process. **(B)** Removal (black diamond), 100 fold (black triangle) or tenfold (black square) dilution of AA in the initial (Incubation 1) BM^−^ incubation decreased the antimicrobial activity of the modified CM compared to CM^R^ (black circle). All bars and points show mean values and error bars represent standard deviation, *N* = 3. Statistical significance was measured by Dunnetts test following two-way ANOVA (*, *P* < 0.05; **, *P* < 0.01; ***, *P* < 0.001).

The above results suggested that it was potentially AA carry over that was causing the antimicrobial effect on *S. aureus* NCTC 6571. This might also hint at why the clinical isolate *S. aureus* 1061 A, shown to be penicillin resistant, was not disrupted by the CM^R^. Hence, the basal medium was supplemented with varying concentrations of the AA solution (0%, 0.01%, 0.1% or 1% (the latter being the manufacturers recommend concentration) v/v), CM^R^ was collected (72 h) and tested for antimicrobial capacity. Test CM (hereafter named CM^T^) which contained 0% AA at any stage of the conditioning process was statistically significantly less antimicrobial activity compared to CM^R^ at concentrations above 12.5% v/v conditioned medium (Fig. 3A; *P* < 0.001 & *P* < 0.01, *N* = 3). At concentrations of 6.25% v/v conditioned medium or below, where the AA concentration is lower due to experimental dilution of the CM^R^ or CM^T^, there was no statistically significant difference between biomass volume in the presence of CM^R^ or CM^T^. This was further supported by diluting out the concentration of the AA used in the initial seeding of the cells (i.e. prior to CM^R^ collection) which demonstrated that at 0.01 and 0.1% v/v AA there was no antimicrobial activity compared to the 1% v/v AA BM control (Fig. 3B; *P* < 0.01, *N* = 3).

### The antimicrobial activity of the 10PCAh CM^R^ is influenced by the choice of tissue culture plastic

**Fig. 4:**
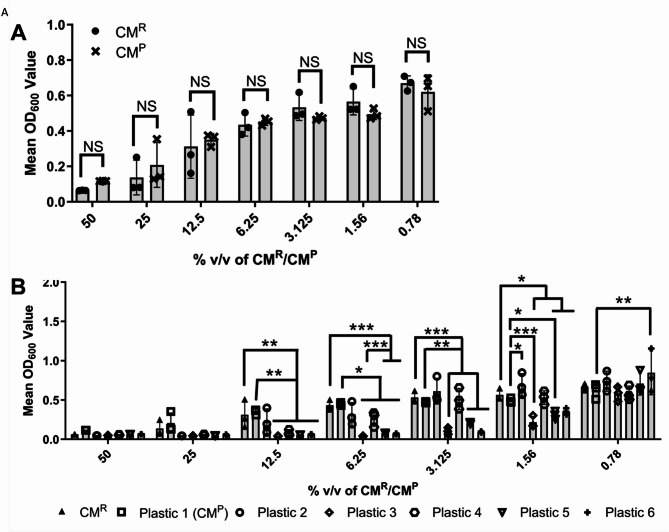
AA binds to the tissue culture surface itself, rather than binding to or being absorbed by cells themselves. **(A)** Cell-free CM^P^ (black cross) demonstrated antimicrobial activity against *S. aureus* NCTC6571 that was statistically indistinguishable from 10PCAh CM^R^ (black circle) (*P* > 0.05). **(B)** The antimicrobial potency of CM^P^ collected within six different commercially obtained plastic tissue culture vessels (P^1^ to P^6^), was assessed using *S. aureus* NCTC 6571 and compared to antimicrobial activity of 10PCAh CM^P^ generated using plastic 1 (white square). Plastic 1 is the plasticware used for all other experiments presented within this manuscript. All bars show mean values and error bars represent standard deviation, *N* = 3. Statistical significance was measured by Dunnetts test following two-way ANOVA (*, *P* < 0.05; **, *P* < 0.01; ***, *P* < 0.001).

Since Fig. 2 demonstrated that cell confluency influenced the potency of CM^R^, we explored the extent to which the observed antimicrobial effect may be due to attachment of the AA to the tissue culture plastic surface, rather than cellular uptake or cell surface attachment. A ‘placebo’ CM was prepared, in which an empty flask was treated as per the routine CM production methodology. The “conditioned medium” collected via this method was named placebo CM (CM^P^). There was no statistically significant difference between the antibacterial activity of the CM^P^ versus CM^R^ against *S. aureus* NCTC6571 (Fig. 4A; *P* > 0.05, *N* = 3). Hence, the presence of cells was not essential to convey the antibacterial activity of the CM^R^. This was further supported by investigating carry over with a variety of tissue culture plastics with various surface treatments, noted by manufacturers to allow various levels of cellular attachment (Table 3; Fig. 4B). All surface treatments were considered proprietary information by the supplier and as such we could not determine the specific treatments the plastics underwent. However, CM^p^ prepared in all six plastics fully inhibited *S. aureus* NCTC 6571 growth at 50% and 25% v/v CM^P^, in a way indistinguishable from CM^P^ prepared in the routinely used tissue culture plastic (P1). At lower concentrations, statistically significant differences in CM^P^ antimicrobial potency were observed (Fig. 4B; *P* ≤ 0.05, *N* = 3). Between CM^P^ concentrations of 12.5% and 1.65% v/v, type 3, 5 and 6 plasticware (P3, P5 and P6 respectively) showed statistically significantly greater inhibition of *S. aureus* NCTC 6571 growth than CM^R^ and CM^P^ (both prepared using plasticware 1). Conversely, plasticware 2 (P2) showed no statistically significant difference in *S. aureus* NCTC 6571 growth inhibition compared to CM^R^ and CM^P^ (prepared in plasticware 1), except at 1.56% v/v where inhibition was statistically significantly less than CM^p^, but not CM^R^ (*P* ≤ 0.05). Overall, no statistical trend was observed in which untreated surfaces created a less potent CM^P^, nor did surfaces advertised as “advanced binding” all show significantly greater potency.

**Table 3:** Details of the plasticwares used and minimum inhibitory concentrations (MIC % v/v) exerted by the CM^P^using different treated surfaces.

Vessel type	Name code	Culture/non-culture surface	Surface Treatment for cells adhesion	Placebo CM (CM^*P*^) MIC%v/v
Cell culture flask T-75	P^1^	Culture PP*	Advanced physical	0.78
Cell culture flask T-75	P^2^		Standard physical	6.25
Cell culture flask T-25	P^3^		untreated	1.56
Screw-capped tube (15 ml or 50 ml)	P^4^	Non-Culture PS**	untreated	6.25
Standard Petri dish 90 mm	P^5^	Non-Culture PP	untreated	1.56
Tissue culture dish 60 × 15 mm	P^6^	Culture PP	Advanced treatment	1.56

*PP = Polypropylene, **PS = Polystyrene.

### The antimicrobial effect is due to the carry-over of penicillin

**Fig. 5:**
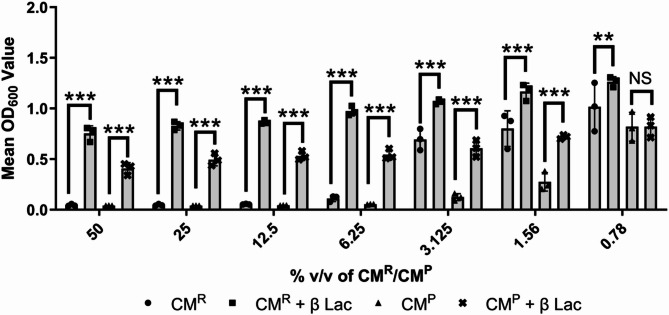
Treatment of CM^R^ and CM^P^ with β lactamase (β-LAC) led to a loss of CM^R/P^ antimicrobial potency against *S. aureus* NCTC6571. CM^R^ (black circle) and CM^P^ (black triangle) were treated with β-LAC to remove penicillin activity (black square and black cross respectively). Statistical comparison of β-LAC treated and untreated CM^R^ and CM^P^ showed a significant reduction in antimicrobial activity following treatment (*P* ≤ 0.01). All bars show mean values and error bars represent standard deviation, *N* = 3. Statistical significance was measured by Dunnetts test following two-way ANOVA (**, *P* < 0.01; ***, *P* < 0.001).

Results suggested that the antibacterial activity of all the tested CM^R^ was actually due to the attachment/adhesion of AA components to the tissue culture plastic surface itself. Furthermore, Fig. 1 demonstrated that this antimicrobial effect was not observed with bacteria that were resistant to penicillin. Hence, neutralizing penicillin (a key component of the routine cell culture medium) was next investigated. When both CM^P^ and CM^R^ were treated with β-lactamase it was possible to remove the antibacterial effect of both CM^R^ and CM^P^ (Fig. 5; *P* < 0.001, *N* = 3) suggesting that penicillin was the key component of the carry-over and it was this, and not cell secreted products, that was responsible for any antimicrobial activity.

## Discussion

Previous work within our group has demonstrated that OMLP-PC cells and their immortalized derivatives, have a contact-independent immunosuppressive effect on lymphocytes^28^ and their EVs can drive wound healing functions, including reducing skin scarring^29^. Furthermore, the OMLP-PC cells and their CM have also been demonstrated to have antimicrobial activity against *Enterococcus faecalis*, *Streptococcus pyogenes*, *Pseudomonas aeruginosa* and *Proteus mirabilis*^27^. Whilst aiming to build further on this work and understand the extent to which the antimicrobial effect of OMPL-PCs was driven by CM-derived EVs, method optimization carried out by the authors led to the discovery of the findings presented within this manuscript.

To determine to what extent the antimicrobial activity of OMLP-PC CM was distinct from other cell populations, additional cell lines were included in this study. The cell lines included were selected as they either represented cell types which would be encountered during a cutaneous wound infection (HaCat, NHh, NIh, NKh, WHh, WIh and WKh) or, to the best of the authors knowledge, would not be expected to have any significant antimicrobial activity (DU145). Surprisingly, CM^R^ from all the cell lines statistically significantly decreased the growth of *S. aureus* NCTC 6571, especially at a CM^R^ concentration of 50% v/v. This is in agreement with previous work showing that antimicrobial peptides (AMPs) secreted by mesenchymal stromal cells were able to inhibit the growth of *S. aureus* ATCC 25,923 as well as *Escherichia coli* ATCC 10,536^30^. Whilst suppression of growth might be expected in the presence of fibroblast, progenitor cell and keratinocyte cell types, it was unexpected that DU145 CM^R^ suppressed *S. aureus* NCTC 6571 growth. The lack of suppression of *S. aureus* 1061 A (penicillin-resistant) was also indicative that a non-AMP inhibition mechanism was responsible since previous studies suggest that AMP activity is independent of the antibiotic resistance profile of bacterial isolates. A recent study^31^ showed that the cathelicidin LL-37 inhibited both growth and biomass formation of MRSA *S. aureus* 1004. Similarly, a recent meta-analysis highlighted that multiple studies report antimicrobial activity of LL-37 against a broad range of the ESKAPE pathogens, including *S. aureus* isolates which are both methicillin-resistant and those with vancomycin-intermediate resistance^32^. Taken together the lack of cell line-specific antimicrobial activity and selective inhibition of only the penicillin-sensitive isolate suggested that a cell-independent antimicrobial mechanism was responsible for the observed effect.

Adding to these initial observations, the SEM images of the *S. aureus* NCTC 6571 isolate in the presence of 50% v/v CM^R^ also appeared to highlight damage to cell walls (Fig. 1C-F). In the presence of CM^R^, a high amount of debris could be observed on the abiotic surface, with some cells appearing to have a “deflated” form (Fig. 1E). This is consistent with previous work^33,34^ in which cell wall integrity loss led to the collapse of *S. aureus* cells, an effect which could be caused by either penicillin or AMP activity. Further investigation of the observed antimicrobial activity of the CM^R^ against *S. aureus* NCTC 6571 highlighted that CM^R^ antimicrobial activity was lost when AA was excluded from the medium used in the initial stages of the conditioning process (CM^T^) (Fig. 3A). Furthermore, the antimicrobial activity was proportional to the exposed (not covered by cell-monolayer) surface area of the tissue culture flasks. We, therefore, hypothesized that the penicillin within the antibiotic supplement was able to bind reversibly to the modified surfaces of the tissue culture plasticware^35,36^. To the best of our knowledge this is the first report of its inadvertent carry over and ‘contamination’ of CM when it has specifically not been used as a supplement in the cell culture medium utilized to create CM.

Although the exact methodology used by each manufacturer to modify the surfaces of their tissue-culturewares is proprietary information, generally, treated surfaces display decreased hydrophobicity, improving protein and cell adhesion^37^. Treatments can either be chemical or physical. In this study, differences were observed in the antimicrobial potency of cell-free CM^P^ produced in several different brands and types of non/tissue-culture flasks although the trends we observed were not necessarily directly linked to the advertised “bindability” of a surface, suggesting that perhaps both the plastic type and surface treatment undertaken influenced AA retention and release. Previous work has also suggested that both the surface wettability and surface energy of tissue-culture plastics can be modified by surface modification^38^. Similarly, the choice of plastic and plasma gas treatment was also shown to be important for biocompatibility of tissue culture surfaces^39^. The authors could not find evidence of previous work indicating that penicillin was able to adsorb onto the polystyrene surfaces used in tissue culture. However, previous work has shown that penicillin G can be adsorbed from complex medium with the use of polymers such as Amberlite XAD-4^40^ and poly(styrene-block-acrylic acid)^41^, with pH, temperature and ethanol concentration all influencing the effectiveness of the adsorption/desorption to the materials.In summary, this work demonstrates that antibiotics can unintentionally be retained on abiotic surfaces used in routine tissue culture and can subsequently be released, contaminating medium with antibiotics which affect cellular function and, directly or indirectly, skew cell-based antimicrobial activity results. Many researchers used PenStrep or AA supplements as part of their routine cell maintenance protocols^22–26,42^, within research relevant to microbial colonization of cells many examples of antibiotic incorporation into growth medium are present^43–50^. Within some of these studies bacterial infection and invasion of assays are performed in the same plasticware in which cells were previously cultured in antibiotic-containing medium^44–48^. While the authors make it clear that when live bacteria are present, medium is switched to an antibiotic-free solution, however, the results presented here show that antibiotics may be retained from the previous medium, being released and potentially influencing bacterial viability and behavior during data collection. Interestingly, several of the studies listed above report antimicrobial activity within their CM or CM-derived products with no identification of known AMPs within the CM^42,43^. As such, there is a potential for AA contamination of CM to contribute to the overall potency of products generated, confounding data and falsely increasing antimicrobial activity. So common is the incorporation of antibiotics into routine TC culture medium that some studies reporting the antimicrobial activity of CM-derived products do not contain information about routine cell culture conditions. For example, a recent study showed that CM derived from bone marrow and umbilical cord stem cells had broad spectrum antibacterial activity, even though LL-37, a potent AMP, was lost during CM nebulization steps^51^. The authors noted that while LL −37 was lost, two other AMPs were still present and this may account for the antimicrobial activity observed. However, since details of the cell growth medium were not included within the manuscript, we cannot be sure that residual antibiotic products, which would also survive the nebulization process, did not contribute to the observed activity. Similarly, mesenchymal stem cells, cultured in an unspecified medium, were shown to have antimicrobial activity against several bacterial pathogens causing gastroenteritis^52^. Within this study, the authors did not identify any potential AMPs within the medium and as the exact composition of the medium is unknown, again, the reader cannot be confident that AA contributed to the observed activity. Indeed, even our own previous research on the antimicrobial properties of CM products noted that antimicrobial activity was present even in the absence of LL-37. As with the previously mentioned study^51^ we identified several alternative AMPs which likely contributed to the antimicrobial activity, however, our inclusion of AA at early stages of cell culture may have led to AA contributing to the activity^27^. In citing these example publications, we make no judgment of the researcher’s methodological choices. Rather these are simply examples selected from the larger body of literature to illustrate the widespread acceptance of antibiotic supplementation for routine tissue culture across many applications. Hence, the authors now recommend that antimicrobial supplementation of medium is carefully considered during development of methods where CM is to be collected for downstream physiological uses. The composition of growth mediums should be recorded in detail within publications and this information should include detailed information on any antimicrobials used, their concentrations and wash steps at each methodological point. Further drug-surface interaction studies are required to fully understand the potential of supplements to bind to the abiotic surfaces used in routine tissue culture. Critically, precise delineation of the antimicrobial mechanism of action of any CM under investigation is an essential step in the validation of future cell-based therapeutics.

## Methods

### Routine maintenance of mammalian cell lines

A full list of the cell lines used within the study is shown in Table 1. All cell lines were routinely maintained in Dulbecco’s Modified Eagle’s medium (DMEM) (Gibco) supplemented with 1% (v/v) 200 mM L-glutamine (Gibco) with or without 10% (v/v) heat inactivated fetal bovine serum (FBS) (Gibco) and with or without 1% (v/v) AA (10,000 U/mL penicillin G, 10,000 µg/mL streptomycin sulfate and 25 µg/mL amphotericin B [Gibco]) at 37 °C in a 5% CO_2_ humidified atmosphere. The two supplemented basal media (BM) are referred to as BM^+^ (with 10% (v/v) FBS and 1% v/v antibiotics/antimycotics) and BM^−^ (without 10% (v/v) FBS and 1% v/v AA) respectively. For routine cell culture, cells were seeded at a density of 4,000 cells/cm^2^ in tissue culture flasks (Sarstedt, UK) in BM^+^ and passaged on reaching approximately 80–90% confluency. Frozen stocks were maintained in liquid nitrogen in BM^−^ supplemented with 50% (v/v) FBS and 10% (v/v) Dimethyl sulfoxide (Fisher).

### Bacterial isolates and inoculum preparation

*Staphylococcus* spp. isolates are listed in Table 1 and were maintained on tryptic soya agar (TSA) (Oxoid) at 37 °C. Following overnight growth of the stocks on TSA, cultures were stored at 4 °C and used repeatedly for up to one month. Bacteria were grown in brain heart infusion (BHI) (Oxoid) broth cultures overnight with shaking at 180 rpm/37°C. The density of the bacterial strains was then adjusted to an optical density at wavelength of 600 nm (OD_600_) of ~ 0.08–0.1. The adjusted culture was then one hundred-fold serially diluted to achieve a cell density of ~ 1 × 10^7^ CFU/ml before use in experiments.

### Production of routine conditioned medium from cell lines

For the generation of routine conditioned medium (CM^R^) the cell lines were seeded into advance-treated (high binding) culture flasks at 4,000 cells/cm^2^ in BM^+^ and incubated at 37 °C with 5% CO_2_ for 48 h. After 48 h, the medium was removed and replaced with BM^−^ and incubated at 37 °C with 5% CO_2_ for a further 72 h. Where washing steps were included, they occurred at the 48 h time point once Incubation 1 medium was removed and prior to the replacement of BM^−^ and subsequent 72 h incubation (Incubation 2). Following the second incubation, cells were at approximately 70–80 confluency, To assess which components within the CM^R^ were responsible for the observed antimicrobial activity, several different conditioning processes were investigated. Full details of these variable factors and the CM^R/P/T^ generation stages at which they occurred are presented within Table 2, with additional information about the plastic surfaces utilized shown in Table 3. Regardless of the preparation method utilized, CM^R/P/T^ was collected and filtered using 0.22 µM PolyEtherSulfone (PES) membranes (Nalgene). After collection, CM^R/P/T^ was either used immediately or aliquoted and stored at −80 °C until use. Once thawed aliquoted CM^R/P/T^ was stored at 2–8 °C and used for up to two weeks.

### Screening the antimicrobial activity of the collected CM^R/P/T^

The Minimum Inhibitory Concentration (MIC) of the collected CM^R/P/T^ was assessed by using microplate turbidimetric growth inhibition. A 50 µl volume of the adjusted bacterial culture, described above, was added to 50 µl of two-fold serially diluted CM^R/P/T^ (diluted in sterile phosphate buffer saline; PBS) in a 96-well flat bottom microtiter plate (Sarstedt, UK). This gave CM^R/P/T^ dilutions of 50, 25, 12.5, 6.25, 3.125, 1.56 and 0.78% (v/v). The plate was incubated at 37 °C for ~ 18 h. to allow bacterial growth. Inhibition of bacterial growth was determined by measuring the turbidity of the wells at OD_600_ post-incubation using a microplate reader (Fluostar Omega). The MIC was considered to be the last well showing no visible growth. MIC was monitored both by inspection visually and by OD_600_. Negative (without bacterial culture) controls of CM^R/P/T^, BHI and PBS were used for background correction. Positive controls of bacterial cultures growing in BHI and/or PBS un-supplemented with CM^R/P/T^ were used for optimum growth comparison.

### Preparation of samples for scanning electron microscopy (SEM)

For assesment of bacterial attachment in the presence of CM^R^, sterile glass coverslips were added to the wells of a 24-well microtiter plate and covered with bacteria at a concentration of 5 × 10^5^ CFU/ml in BHI supplemented with either 50% (v/v) CM^R^ or 50% (v/v) BM^−^. Cultures were incubated statically overnight at 37 °C with 5% CO_2_ to allow biomass formation. Following incubation, coverslips were washed once with sterile PBS to remove loosely attached bacterial cells and fixed using 2.5% (v/v) glutaraldehyde (Sigma). Samples were dehydrated by incubation in increasing concentrations of ethanol (50, 70, 90, 100% (v/v) respectively) (Fisher), followed by serial incubation in hexamethyldisilazane (HMDS) (Merck) diluted in ethanol (ratios of 1:2, 2:1 and HMDS only respectively). Excess HMDS was left to evaporate before sputter coating using a K650x sputter coater (Quorum Technologies) and imaging with a Tescan Vega 3 (Tescan Ltd). For each cover slip, images at low and high magnification were taken at each of the cardinal compass points and in the center of the disc to ensure each sample was representatively imaged.

### Determination of protein concentration

OD_600_ 0.9 adjusted cultures of *S. aureus* NCTC 6571 and *S. aureus* 1061 A, in either sterile PBS or PBS containing 50% (v/v) CM^R^, were prepared as described above. The cultures were incubated statically overnight at 37 °C with 5% CO_2_ before centrifugation at 10,000 x*g* for 10 min to pellet bacterial cells and debris. The bacterial supernatant was removed and protein concentration was determined using the Pierce BCA Protein Assay Kit (ThermoFisher) according to the manufacturer’s instructions.

### Beta-lactamase hydrolysis activity assay

The AA used in the maintenance of the cells contained penicillin G sodium (10000 U/ml). Penicillin G activity was removed from the CM^R/P^ by adding a broad-spectrum mixture of beta lactamase (β-Lac) enzymes (Oxoid, UK). Each 1 U of β-Lac mixture hydrolysed 1 µmole of the penicillin G. The MIC of the β-Lac-treated cell culture medium relative to the untreated medium was screened against *S. aureus* NCTC6571. Briefly, β-Lac mixture powder was freshly prepared in sterile water. One ml of β-Lac mixture was equivalent to 60,000 Levy units of β-Lac-I and 6,000 Levy units of β-Lac-II. This was added to 5–10 ml of the CM^R^ or CM^P^ directly before monitoring their MIC against *S. aureus* NCTC6571.

### **Statistical analysis**

All experiments were at least three biologically independent replicates. Analysis of data was carried out using GraphPad Prism software (version 5.0-prism.exe). All data was determined to be parametrically distributed using Shapiro-Wilk test. Numerical data were expressed as Mean with error bars showing standard deviation. Comparison between 3 groups was undertaken using the parametric two-way ANOVA then a post hoc-tests was used for pair-wise comparison. Statistical test names are included within figure legends. All tests were two-tailed. A *p-value* ≤ 0.05 was considered significant.

## Supplementary Information

Below is the link to the electronic supplementary material.

Supplementary Material 1

## Data Availability

The datasets used and/or analysed during the current study are available from the corresponding author upon reasonable request.
