# Supplementary material for: Antibiotic carry over is a confounding factor for cell-based antimicrobial research applications
Source: Sci Rep. 2025 Aug 3;15:28310. doi: 10.1038/s41598-025-14186-7 (PMC12319067; doi:10.1038/s41598-025-14186-7)
Supplement: Supplementary file 1 — Supplementary Material 1 [file 41598_2025_14186_MOESM1_ESM.pdf]

Supplementary Figure Legend

Supplementary Figure 1: CM^R antimicrobial activity is highest in penicillin sensitive *Staphylococcus* spp. isolates. Overnight growth of multiple *Staphylococcus* spp isolates in the presence of 50 %, 12.5 %, 3.13 % or 0.78 % v/v CM^R from nine cell lines was determined. *Staphylococcus* spp isolates are as follows: **(A)** *S. aureus* 1004A (penicillin resistant), **(B)** *S. aureus* 1038B (penicillin resistant), **(C)** *S. aureus* NCTC 4137 (penicillin sensitive), **(D)** *S. aureus* NCTC 7791 (penicillin sensitive), **(E)** *S. aureus* EMRSA-15 (penicillin resistant), **(F)** *S. epidermidis* 1064A (penicillin resistant), **(G)** *S. epidermidis* ATCC 12228 (penicillin sensitive), **(H)** *S. warneri* NCTC 5955 (penicillin sensitive). All bars show mean values and error bars represent standard deviation, N = 3. For each bacterial isolate, the growth in CM^R at each % v/v concentration was statistically compared to the bacterial growth achieved in the same concentration of BM^-. Statistical significance was measured by Tukey test following one-way ANOVA (*, $P < 0.05$; **, $P < 0.01$; ***, $P < 0.001$). SEM images are representative, scale bar = 10 µM.

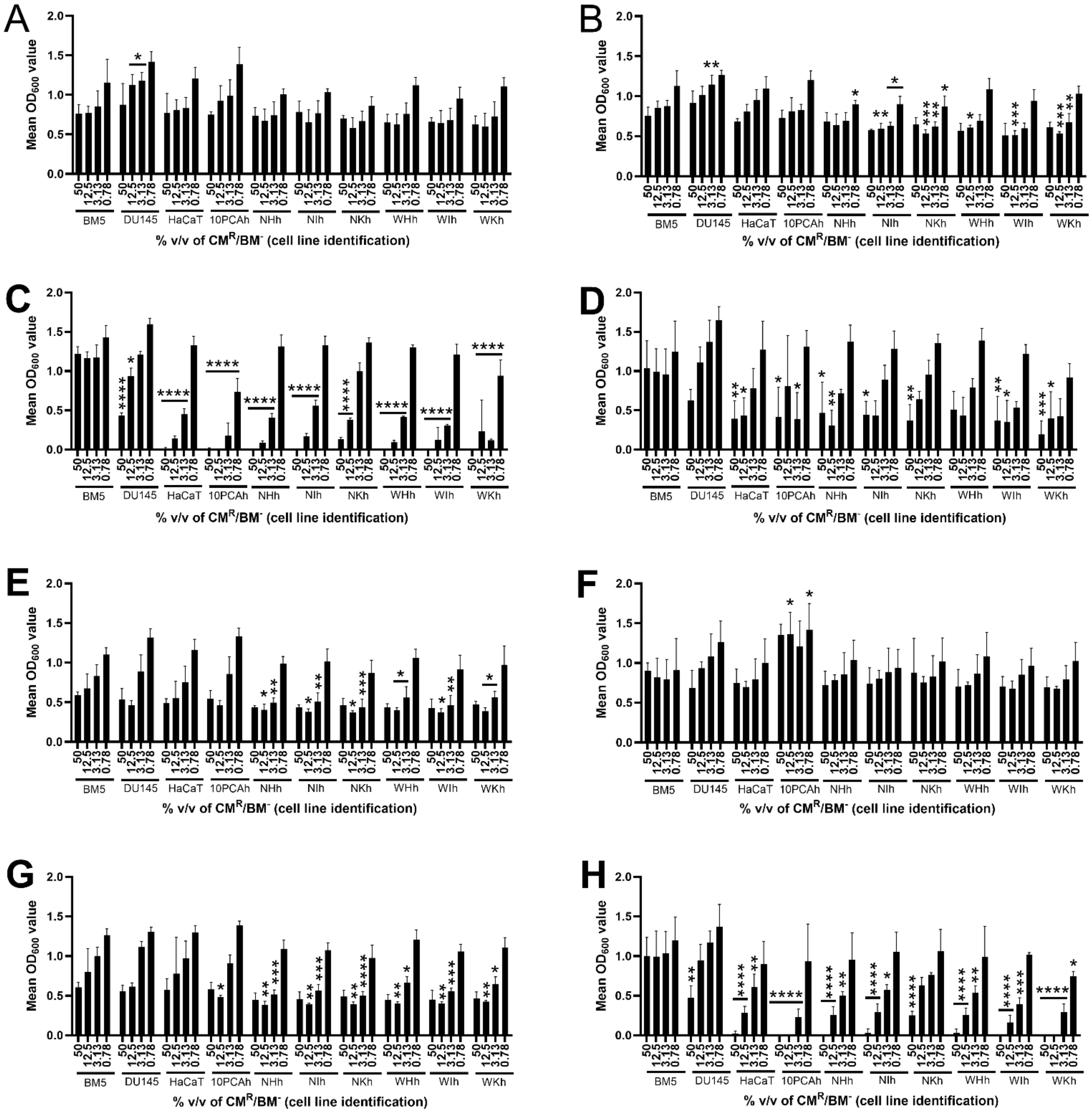
A
B
C
D
E
F
G
H
Mean OD600 value
2.0
1.5
1.0
0.5
0.0
50
12.5
3.13
0.78
BM5
DU145
HaCaT
10PCAh
NHh
NIh
NKh
WHh
WIh
WKh
% v/v of CMR/BM- (cell line identification)
